# Pantoprazole and other proton pump inhibitors, sleep, and cardiovascular safety: weak but plausible evidence

**DOI:** 10.1590/1806-9282.20260216

**Published:** 2026-08-24

**Affiliations:** 1 Instituto de Assistência Médica ao Servidor Público Estadual de São Paulo – São Paulo (SP), Brazil.; 2 Universidade Federal de São Paulo, Escola Paulista de Medicina – São Paulo (SP), Brazil.; 3 Associação Médica Brasileira – São Paulo (SP), Brazil.; 4 Sleep Institute/Associação Fundo de Incentivo à Pesquisa – São Paulo (SP), Brazil.; 5 Faculdade de Medicina do ABC – Santo André (SP), Brazil.

## INTRODUCTION

Nearly a quarter of adults will use a proton pump inhibitor (PPI) in their lifetime^1^. These drugs are widely prescribed to suppress gastric acid secretion in the management of peptic diseases (dyspepsia, gastroesophageal reflux disease, ulcers) and are generally regarded as having a highly favorable safety profile. Nevertheless, adverse effects may occur, either as indirect consequences of acid suppression, leading to impaired nutrient absorption or bacterial overgrowth, or as direct or idiosyncratic drug reactions, including kidney injury^2^. An increased risk of cardiovascular disease has been suggested as a potential adverse effect, although systematic reviews have reported inconsistent findings^3^. Sleep disturbances have been reported in patients treated with PPIs^4^. However, in individuals with sleep disorders, particularly obstructive sleep apnea, who frequently present with gastroesophageal reflux, acid suppression therapy has consistently been reported to improve sleep quality by reducing nocturnal reflux events, even though apnea–hypopnea indices generally remain unchanged^4-6^. Given the strong links between poor sleep, hypertension, metabolic dysregulation, and cardiovascular disease, this pathway may represent an additional, indirect mechanism through which long-term PPI therapy influences cardiovascular outcomes. Here, we discuss the arguments for and against the potential role of PPI and cardiovascular disease.

### Pro arguments

PPI may damage the cardiovascular system by multiple mechanisms.

**Figure 1:**
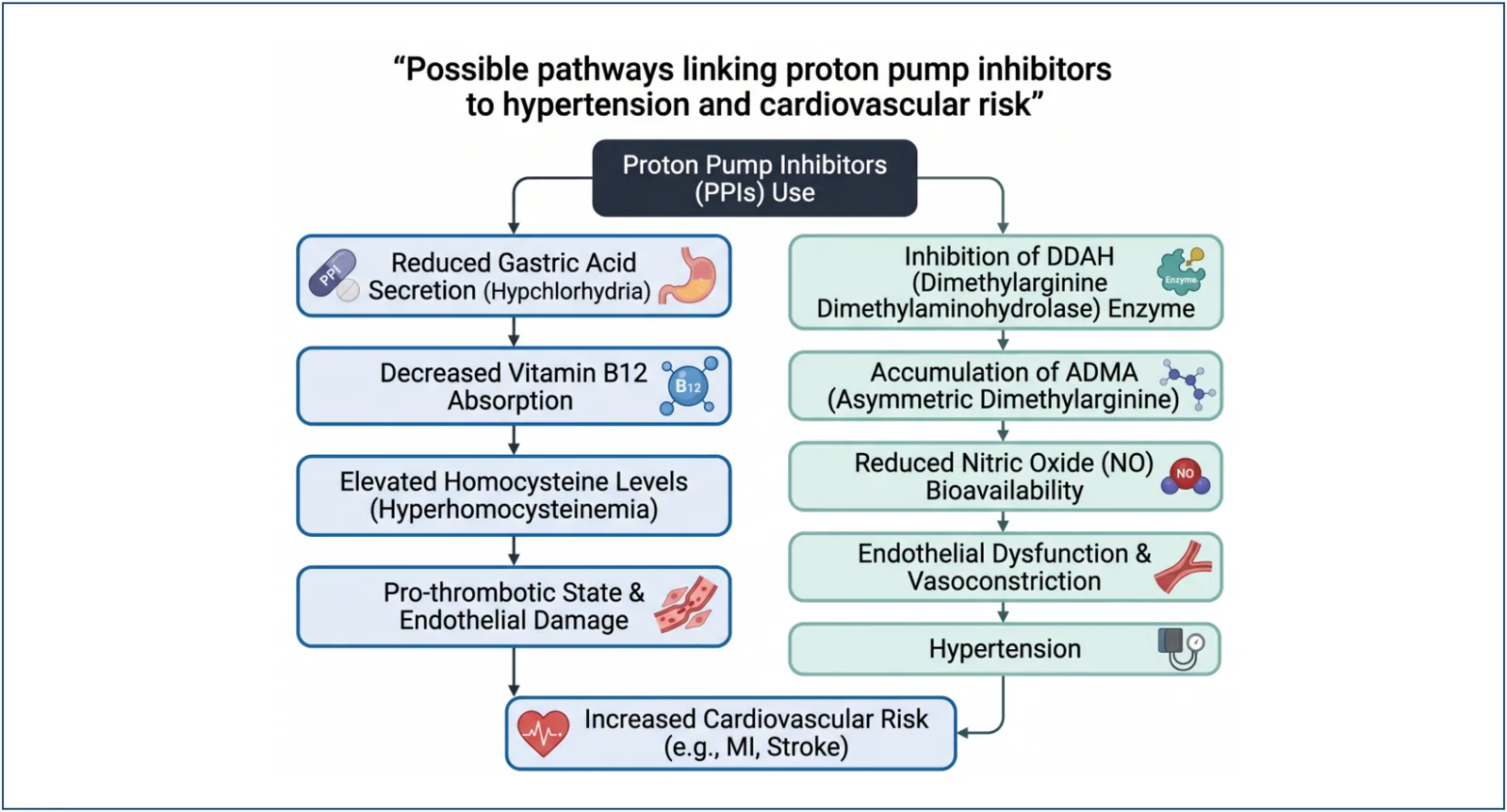
Possible pathways linking proton pump inhibitors to hypertension and cardiovascular risk.

PPI may cause hypertension. PPI may cause hypertension by decreasing vasodilatation and increasing vasoconstriction. This is mediated by reducing bradykinin-induced nitric oxide (NO) generation and endothelial NO synthase (eNOS)^1^. Omeprazole decreases the formation of prostaglandin I_2_ metabolites, particularly 6-keto-prostaglandin 1α. Kamiya et al. suggest that PPIs reduce NO availability, most likely through a previously suggested mechanism (e.g., decreased eNOS production or increased intracellular asymmetric dimethylarginine (ADMA) level) (Figure 1)^7^. PPIs have this effect through inhibition of dimethylarginine dimethylaminohydrolase 1 (DDAH1), which normally degrades ADMA; the resulting accumulation increases the risk of cardiovascular disease and mortality by promoting vasoconstriction^3,7^.

PPI may damage the myocardium. Santos et al. demonstrated that omeprazole, immediately before cardiac ischemia and reperfusion, in a rat model, increased serum concentrations of cardiac injury markers, as well as the incidence of atrioventricular block, with a lethality of 100%, probably due to decreased NO production^8^.

**Figure 2:**
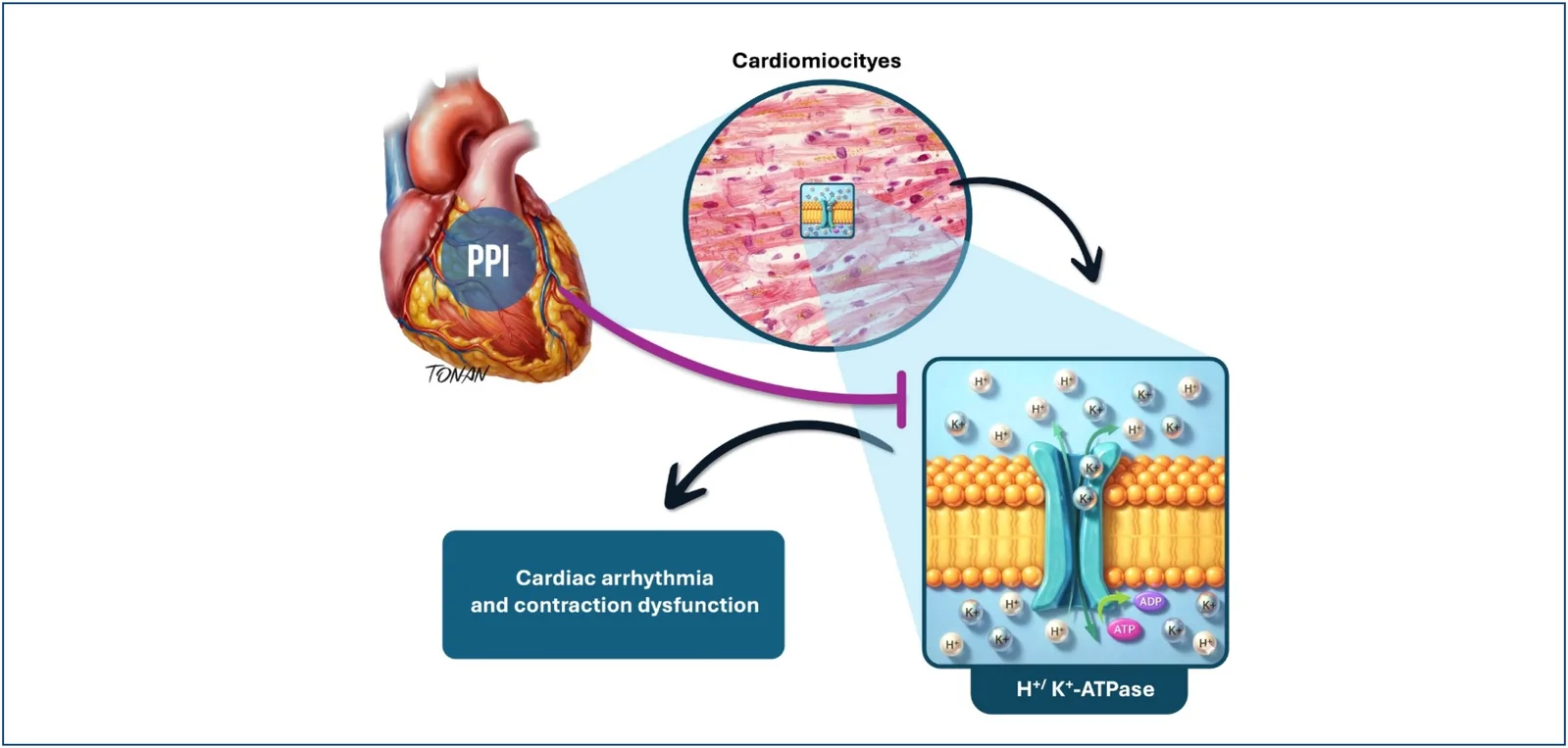
Inhibition of the H+/K+-ATPase proton pump present in cardiomyocyte membranes and the resulting effects of this mechanism: cardiac arrhythmias and contraction dysfunction. PPI: proton pump inhibitors.

PPI may decrease myocardial contractility. Recently published data reported the expression of both H^+^/K^+^-ATPase mRNA and protein in human and rabbit cardiac tissue^9^. There is physiological and biochemical evidence for a myocardial H^+^/K^+^-ATPase^9^. Schillinger et al. proposed that the enzyme could play an important role in the control of cardiac K^+^ and H^+^ homeostasis in rat hearts (Figure 2)^9^. Thus, cellular acidosis, which is known to reduce cardiac contractility mainly at the level of myofilament response to intracellular [Ca^2+^], could be induced by inhibition of H^+^/K^+^-ATPase^10^.

**Figure 3:**
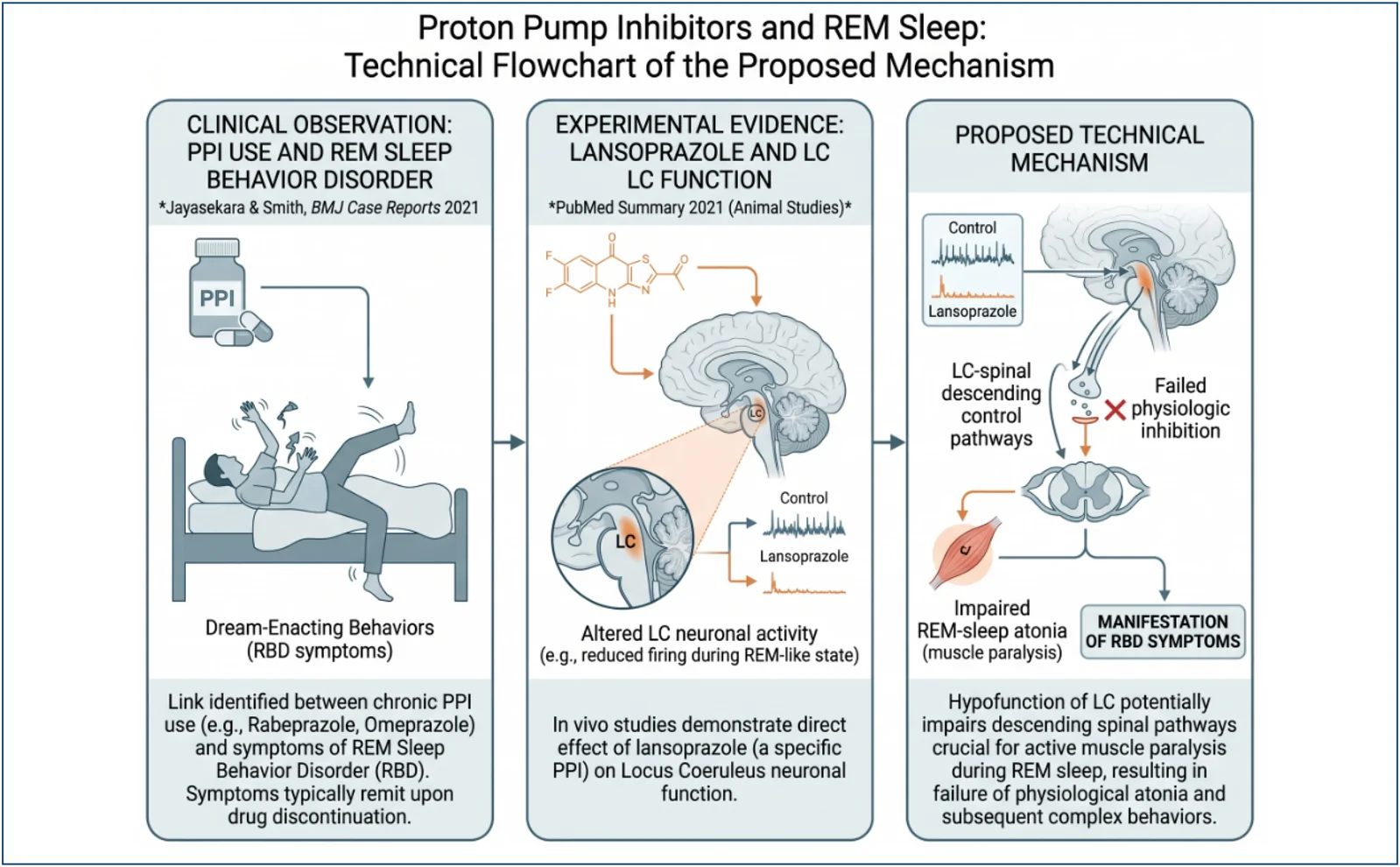
Proton pump inhibitors and rapid eye movement sleep: technical flowchart of the proposed mechanism^13,14^. PPI: proton pump inhibitors; LC: locus coeruleus; REM: rapid eye movement; RBD: REM Sleep Behavior Disorder.

PPI and sleep disturbances. Beyond direct vascular and myocardial mechanisms, PPI use has also been linked to sleep disturbances, which are independently associated with hypertension, metabolic dysregulation, and cardiovascular risk^6^. Patients with obstructive sleep apnea frequently present with gastroesophageal reflux, and in this subgroup, PPI therapy has been shown to improve sleep quality, although without significant changes in apnea–hypopnea indices^6^. Experimental evidence in animal models indicates that proton pump inhibition with lansoprazole significantly enhances rapid eye movement (REM) sleep duration, likely via modulation of CO_2_-dependent pH homeostasis (Figure 3)^11^. Clinically, a case report describes a patient diagnosed with REM sleep behavior disorder whose symptoms resolved upon discontinuation of omeprazole and recurred with lansoprazole, suggesting PPI exposure can influence REM sleep regulatory mechanisms (Figure 3)^12^. These findings suggest that sleep disruption may represent an indirect mediator between PPI exposure and adverse cardiovascular outcomes, warranting further investigation.

### Con arguments

Ghebremariam and coworkers described that in patients with coronary disease, compared to healthy volunteers, PPI usage had no discernible effect on vascular endothelial function by measuring serum levels of ADMA^13^. These findings made the authors wonder if DDAH1 inhibition is a clinically significant mechanism for elevated cardiovascular risk associated with PPI use^13^. A common high-dose regimen of pantoprazole (80 mg over 2 min and then 8 mg/h intravenously) did not cause clinically significant impairment of hemodynamics and left ventricular function in healthy volunteers^14^.

Recent data indicate that the population incidence of PPI use is rising, while cardiovascular mortality appears to be declining. We argue, however, that conditions including ischemic and hemorrhagic strokes and pulmonary thromboembolism should be classified as cardiovascular rather than neurological or pulmonary diseases. When these outcomes are taken into account, the burden of cardiovascular disease continues to increase, and it remains the leading cause of death worldwide. Moreover, PPIs have been implicated in several pathophysiological processes that may affect the cardiovascular system, including reduced absorption and altered metabolism of antiplatelet agents from gastric alkalinization, inhibition of hepatic microsomal enzymes, lower vitamin B12 levels, and consequent elevations in homocysteine (Figure 1)^15^. Notably, most studies addressing PPI safety have not evaluated sleep-related pathways, which remain largely unexplored despite their potential cardiovascular relevance.

### Alternatives to proton pump inhibitor

Other pharmacological classes, older and newer than PPI, are also gastric acid blockers. The effect of these drugs on the cardiovascular system has been poorly studied.

Histamine H2 blockers. Cardiovascular adverse effects are incredibly uncommon and unexpected with typical dosages of ranitidine (at most 1 in 1 million individuals). They mainly consist of atrioventricular blockades and sinus bradycardia. Clinical trials have not demonstrated a substantial pharmacological effect of ranitidine on the cardiovascular system via H2-receptors^16^. Antibodies have been used to identify H1 and H2 receptors in the human heart^17^. Human atrial samples express more H1 than H2 receptors^17^. However, the histaminergic positive inotropic and chronotropic effects in the human atria are now believed to be caused by the H2 receptors^18^, and H2 receptor antagonists like cimetidine and famotidine may prevent the positive inotropic effects of histamine^19^. Hence, with typical oral dosages of ranitidine, cardiovascular adverse effects are incredibly uncommon and unexpected. They mainly consist of atrioventricular blockades and sinus bradycardia, which go away as the medication is stopped, especially after quick intravenous infusion. Although individual sensitivities cannot be excluded in a few isolated instances, clinical trials have not indicated a substantial pharmacological effect of ranitidine on the cardiovascular system via H2-receptors^20^. With regard to sleep, H2 receptor antagonists may provide partial relief of nocturnal reflux and thereby improve sleep quality in patients with mild gastroesophageal reflux disease, although robust evidence from sleep studies is lacking^5^.

Potassium-competitive acid blockers (BPAC). There are no studies proving the safety of BPAC (vonoprazan) in patients with cardiovascular diseases. Sasaki et al. affirmed that their use was able to prevent upper gastrointestinal bleeding associated with gastric acidity in patients undergoing percutaneous coronary intervention (post-PCI) or antithrombotic pharmacological treatment, which is why we believe that BPAC can be considered an interesting option for patients with cardiovascular disease who require agents that reduce gastric acidity^21^. No data is currently available on the impact of potassium-competitive acid blockers on sleep physiology or sleep-related reflux symptoms, representing an open field for future investigation.

Non-pharmacological strategies also represent promising approaches for addressing nocturnal reflux and sleep impairment. Lifestyle interventions, such as head-of-bed elevation, light evening meals, and weight reduction, have been shown to reduce nocturnal gastroesophageal reflux and improve sleep quality. In patients with the common coexistence of obstructive sleep apnea and reflux, the combined use of continuous positive airway pressure and acid-suppressive therapy (either PPI or H2 receptor antagonists) may be more effective in restoring normal sleep architecture than pharmacological treatment alone^22,23^.

## CONCLUSION

We conclude that the evidence for PPI as a risk factor for cardiovascular disease is weak, but probable, and the alternatives are not fully studied. H2 blockers are less efficient compared to PPI in inhibiting acid secretion, but it is reasonable to use them in patients at risk for cardiovascular disease and non-severe gastrointestinal disorders. BPAC seems to be more efficient when compared to PPI in acid blockage, but its cardiovascular risk has not entirely been addressed. In addition, the potential impact of PPIs on sleep deserves further attention, particularly in patients with gastroesophageal reflux disease or obstructive sleep apnea, where acid suppression may improve sleep quality yet also interact with mechanisms relevant to cardiovascular health.

## Data Availability

The datasets generated and/or analyzed during the current study are available from the corresponding author upon reasonable request.
